# From Research to Practice: Implementing an Evidence-Based Intervention for Nurse Well-Being in a Healthcare System

**DOI:** 10.3390/healthcare13182369

**Published:** 2025-09-20

**Authors:** Amanda K. Bailey, Hong Tao, Amanda T. Sawyer

**Affiliations:** AdventHealth Research Institute, 800 N Magnolia Ave, Suite 500, Orlando, FL 32803, USA; hong.tao@adventhealth.com (H.T.); amanda.sawyer@adventhealth.com (A.T.S.)

**Keywords:** burnout, nursing, caregiver support interventions, holistic well-being

## Abstract

**Background:** In response to the high prevalence of burnout in nursing, a hospital research team developed, studied, and implemented RISE (Resilience, Insight, Self-Compassion, Empowerment), a novel psychoeducational group program designed to reduce distress and promote well-being among professional caregivers, specifically nurses and nurse leaders. Pilot studies and randomized controlled trials showed positive results, and thus, the program was operationalized. **Methods:** This quality improvement/quality assurance (QI/QA) project involved scaling the program and gathering data to evaluate implementation and impact on well-being indicators. The intervention involves structured weekly (8–9 weeks) 90-min group sessions integrating mindfulness, cognitive-behavioral techniques, and acceptance and commitment therapy. Eight licensed mental health providers were trained and delivered the program. Implementation outcomes included adoption/stakeholder engagement, fidelity, provider satisfaction, participant engagement, and sustainability. Participant outcomes were measured through validated scales and participant feedback forms. The implementation process was examined at the participant, provider, and organizational levels to identify barriers and enabling factors. **Results:** The program was implemented in seven acute care hospitals. From January 2023 to December 2024, 160 participants completed the program. Effective implementation strategies included intensive training and supervision of qualified providers, multi-departmental collaborations, and rigorous fidelity monitoring. Quality improvement processes addressed challenges such as early attrition and administrative burden. Evaluation data from pre- and post-intervention surveys demonstrated statistically significant improvements in psychological outcomes, with high satisfaction reported by both participants and providers. **Conclusion:** The findings support the effective implementation of the program as part of a broader organizational strategy to address nurse burnout and workforce mental health. Lessons, implications, and future directions for healthcare leaders are discussed.

## 1. Introduction

Translating psychosocial research interventions into real world settings is a complex process that requires both an evidence-based intervention and an implementation strategy [1,2]. Within a healthcare system, it requires a multi-faceted, multi-departmental approach, involving highly engaged stakeholders and broad organizational support. While it is customary to investigate and report on the implementation of evidence-based healthcare practice for patient care, this project provides a novel perspective on how to integrate well-being programming for professional caregivers, such as nurses and unit nurse leaders. Using conceptual and theoretical frameworks from implementation literature combined with operations knowledge from key organizational stakeholders, this paper describes an approach to scale an evidence-based psychoeducational group program in a multi-campus healthcare system utilizing a quality improvement/quality assurance design (QI/QA).

Well-being programming should align with broader labor practice frameworks in healthcare, specifically those that address the humanness components, including well-being, self-efficacy, and empowerment, in addition to contextual issues, such as staffing, training, and quality of care. For example, the Human-Centered Health Workforce Framework proposed by Nwankwo and colleagues [3] illustrates the functional roles of clinicians in the healthcare system while highlighting their unique values and attributes. This framework demonstrates the need to care for healthcare workers’ holistic well-being when considering human resource management practices and outcomes to ensure fulfillment, satisfaction, and meaning in work. This novel psychoeducational program focuses on the whole person, not just the professional.

### 1.1. Nurse Well-Being

Nursing can be an emotionally and physically draining field of work, leaving individuals fatigued and overwhelmed. Burnout and work stress can interfere with relationships, diminish joy and satisfaction, and disrupt connection to purpose. Burnout often afflicts professional caregivers and is characterized by emotional exhaustion, depersonalization, and reduced personal accomplishment [4]. As formal caregivers, nurses are vulnerable to burnout due to a combination of chronic stress, exposure to suffering, and a lack of resources, with insufficient opportunity to rest and renew energy [4]. Emotional and psychological effects may include anger, anxiety, disillusionment, fear, frustration, hopelessness, and lack of empathy. Physical effects may include fatigue, gastrointestinal problems, headaches, insomnia, and muscle tension [5,6,7]. A significant development in burnout research suggests that symptoms of burnout may be manifestations of traumatic stress [8], making it necessary to have treatment approaches that address the overall mental health of nurses, not only work stress. Furthermore, for professional caregivers, there might be an accumulation effect due to caregiving in other roles (e.g., informal caregiver of a spouse or parent), leading to additional burden and higher burnout levels [9]. Many nurses are double or even triple-duty caregivers, highlighting the significant risk of emotional exhaustion and mental health impairment.

The impact of stress injuries extends beyond personal consequences. Traumatic stress, burnout, and psychological suffering of nurses and nurse leaders can decrease productivity and negatively affect the quality of care [9]. These struggles can have negative effects on staff retention, patient satisfaction, perceptions of safety, competent safety behaviors, and job satisfaction and can lead to higher clinical errors [7,10,11,12]. Burnout costs the healthcare system an estimated $4.6 billion a year, and that number increased during the pandemic [13]. In a large national sample of nurses who left their jobs, over 30% cited burnout as the reason for their voluntary separation from work, with many attributing their burnout to understaffing and stressful work environments [14].

The COVID-19 pandemic highlighted and intensified the existing mental health ramifications of working in healthcare [15]. Nurses and other healthcare workers have increased risks of depression, anxiety, acute stress reaction, post-traumatic stress disorder, insomnia, and burnout [15,16,17]. Unit-based nurse leaders faced unprecedented challenges related to work overload, competing priorities, financial responsibilities, constant change, lack of resources, and low staffing [18]. A survey conducted by the American Organization for Nursing Leadership showed an increase in the percentage of nurse leaders listing “mental health and well-being of staff” as a top concern from 17% in April 2020 to 67% in February 2021. Caring for the mental health and well-being of nurses remains an imperative.

Efforts to address burnout and workplace stress have historically focused on individual-level educational programs that teach stress management and resilience skills, for example [19,20,21,22]. In practice, these individual-level interventions tend to be variable in duration and objectives and often lack a rigorous protocol for intervention delivery. Furthermore, there is scant literature exploring the operationalization of well-being interventions for nurses once their efficacy and/or effectiveness have been established.

Translating evidence-based interventions into clinical settings is an investment in healthy workplace culture and requires organizational commitment [22,23,24]. Healthcare leadership would benefit from packaged, protocolized programs that can be practically integrated into their employee health strategy. Best practice entails developing interventions systematically using evidence and theory, testing feasibility in pilot studies, and then using exploratory and rigorous evaluations [25]. The results should then be disseminated, and subsequent research can assist and monitor the process of implementation [25,26]. This established guidance for developing and evaluating complex interventions was followed throughout this QI/QA project.

### 1.2. Program Development and Previous Studies

In response to the prevalence of high burnout and a growing need for support of hospital nurses in 2018, a research team within a multi-campus healthcare system developed a program called RISE, which is an acronym for Resilience, Insight, Self-compassion, and Empowerment. It is a psychoeducational group intervention, which aims to promote self-care, reduce stress and burnout, and improve indicators of well-being. The program involves weekly 90-min group sessions led by a licensed mental health professional (LMHP) over the course of 8 to 9 weeks, with 6–8 participants per group cohort.

**Figure 1:**
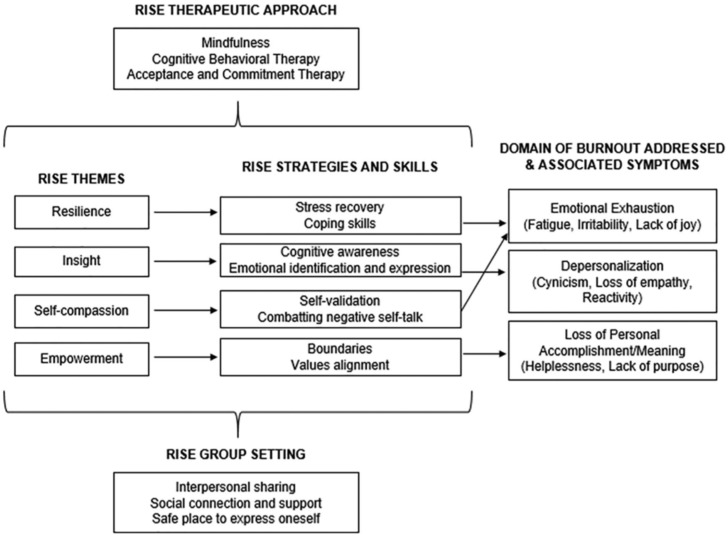
Conceptual Process Model of the Intervention.

The rationale, theoretical framework, and development of the program were previously published [27]. Briefly, participants learn knowledge and skills that are applied to all areas of life while processing the emotional impact of their work. Combining education with the therapeutic process and support can have a synergistic and sustainable impact on participants’ change motivation and healing. The program’s conceptual process model, shown in Figure 1, provides the foundation for the group process, content delivery, and skill development [27]. The therapeutic approach is derived from the evidence-based practices of acceptance and commitment therapy (ACT) and cognitive behavioral therapy (CBT), with an emphasis on mindfulness [27].

A pilot study conducted in 2019 confirmed the feasibility and acceptability of the 8-week program in hospital-based direct care nurses. The results of a randomized controlled trial (RCT) showed decreases in burnout (i.e., emotional exhaustion) and perceived stress scores and increases in resilience, stress mindset, self-compassion, and insight scores [28]. Furthermore, RISE showed promise as a strategy to support retention among nurses. A matched case-control study found retention during the pandemic to be 20% higher among nurses who had participated in RISE compared to nurses who had not [29].

The program was adapted for unit-based nurse leaders (i.e., nurse managers, assistant nurse managers) during the COVID-19 pandemic. Work overload, low work–life balance, competing priorities, financial responsibilities, managing staff conflicts, and lack of support contribute to the development of burnout among nursing leaders [30]. In addition, during the pandemic, nurse managers were responsible for providing psychosocial support to nurses while managing their own family needs, stress, and exhaustion [31]. Considering unit-based nurse leaders’ scope of influence on patient outcomes, nurse job satisfaction, and organizational culture, there is scant research on interventions that support their own mental health and professional wellbeing [31].

The most noteworthy change in RISE for nurse leaders was from in-person to virtual delivery. Also, within the context of the pandemic, constructs of post-traumatic growth (PTG) and professional quality of life were included as additional aims. PTG describes the positive psychological change resulting from the process of moving through adversity, traumatic events, or crises [32]. Importantly, growth emerges from the process of coping and overcoming, not from the trauma itself. Literature suggests that PTG can occur in one or more of the following areas: appreciation of life, relationships with others, new possibilities in life, personal strength, and spiritual change [32].

The program for leaders was 9 weeks due to the addition of leadership principles and PTG in the curriculum. A pilot study confirmed the feasibility and acceptability of the adapted program (i.e., RISE for Nurse Leaders) [30], and an RCT was conducted in 2021 and showed significant improvement in post-traumatic growth, self-reflection and insight, self-compassion, psychological empowerment, and compassion satisfaction, as well as significant reductions in perceived stress, burnout, and secondary traumatic stress [31]. A qualitative analysis found that the program enabled participants to prioritize self-care, contributed to participants feeling empowered to make positive changes in their work and home lives, and fostered a sense of connection and belonging [33]. Importantly, participants also expressed a perceived improvement in their ability to be effective leaders [33].

The RISE program, as a comprehensive approach, is gaining support as an empirically supported intervention (ESI) [2]. Evidence-based practice is defined by the American Psychological Association (2005) as the integration of the best available research with clinical expertise [34]. One approach to advancing the use of evidence in practice is the direct application of ESIs. ESIs are tested under scientific rigor and show consistent positive results in different trials [2]. The direct application of ESIs involves integrating interventions that have some evidence for their efficacy and effectiveness for a given population (e.g., nursing) or clinical problem (e.g., burnout) into routine settings [2]. Collaboration between researchers, LMHPs, and hospital leadership has led to the systematic translation of RISE from research to practice.

This paper discusses the processes and methods for implementing an evidence-based psychoeducational group program for the nursing population. Processes based on theory, scientific, and operational knowledge were used to identify and address barriers to implementation effectiveness and to enhance enablers for uptake. The implementation process was examined at the participant, provider (i.e., RISE facilitator), and organizational levels to identify factors related to adoption, delivery, fidelity, and sustainability.

## 2. Materials and Methods

### 2.1. Design

**Figure 2:**
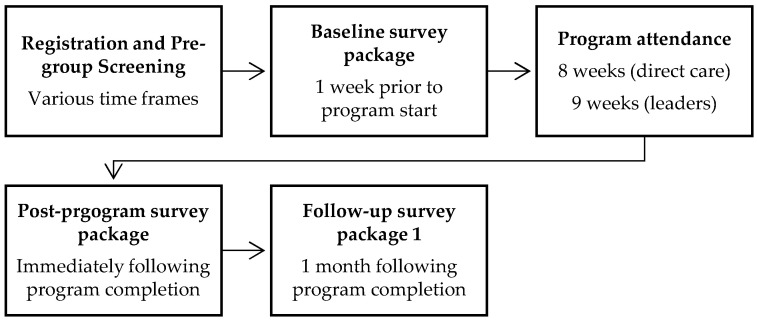
Study timeline for participants.

This project was approved by the Institutional Review Board (IRB) and determined to be quality improvement/quality assurance and was not research on human subjects. The project involved scaling the RISE program to hospital campuses and gathering data to evaluate program implementation and determine its impact on participant well-being indicators. Program participation was voluntary, and participants had to consent to both attendance and evaluation activities. Nurses, assistant nurse managers (ANMs), and nurse managers (NMs) were invited to enroll in the program through marketing materials. To eliminate issues related to power differentials among participants, group offerings were separated into distinct cohorts for direct care nurses, ANMs, and NMs. Participants who registered for RISE were able to self-select into the study project (non-randomized), and quantitative data was collected from those study volunteer participants (n = 50). Figure 2 shows the timeline of study activities for participants.

### 2.2. Setting and Context

Program operationalization took place at a multi-campus faith-based healthcare system headquartered in Florida. Seven facilities supported the rollout of the program on their campuses. Eight LMHPs were trained as program facilitators. These individuals marketed and enrolled nurses and unit-based nurse leaders from their designated facility. Potential participants attended a pre-group screening with the facilitator, signed an informed consent document, attended the program, and completed an anonymous feedback form at the end of the program. Pre-implementation phases began in December 2021, with program launch occurring for nurses and unit-based nurse leaders in January 2023 and June 2023, respectively. This project utilized a rolling recruitment and enrollment approach.

### 2.3. Data Collection

Data collection occurred in two areas: implementation (i.e., adoption, training, fidelity, provider satisfaction, sustainability) and participant outcomes (i.e., satisfaction feedback forms, self-report scales). Quantitative data was collected at the two timepoints of baseline (i.e., pre-program) and endpoint (i.e., post-program) through an electronic data capture system, Microsoft Forms, sent through email links. Using convenience sampling, data was collected between January 2023 and December 2024.

The following validated scales were used to measure the impact of the program on well-being: Brief Resilience Scale [35], where higher scores indicate greater resilience; Self-Reflection and Insight Scale [36], where higher scores reflect more self-reflection and insight; Self-Compassion Scale—Short Form [37], where higher scores denote greater self-compassion; the Psychological Empowerment Instrument [38], where higher scores represent stronger feelings of empowerment; Perceived Stress Scale [39], where higher scores signify more perceived stress; Posttraumatic Growth Inventory [40], where higher scores are reflective of more positive psychological growth following adversity or crisis; General Self-Efficacy Scale [41], where higher scores indicate stronger belief in one’s ability to succeed at specific tasks; and the Maslach Burnout Inventory [42], where higher scores in emotional exhaustion and depersonalization reflect greater burnout, while higher scores in personal accomplishment reflect lower burnout. In addition, demographic questionnaires were collected, which included participant age, gender, race, ethnicity, job title (i.e., direct care nurse, nurse leader), and years of experience.

Participant feedback was collected after program completion through an anonymous form. The form was created by the program developer and the principal investigator of the research studies. It asked about the usefulness of the program components of learning and self-reflection, active discussion, mindfulness practices, experiential activities, skill development, and support [27]. It also allowed open-ended responses to question prompts about aspects of the program participants would keep the same or change, what they plan to change as a result of program attendance, and the perceived impact of the program on their well-being and daily life.

### 2.4. Implementation

Implementation is described as a set of activities designed to put into practice a treatment program of known dimensions [43]. There are several theories and models that outline the best way to do this. An approach to this process includes phases such as dissemination (i.e., how well information about the program is provided to communities), adoption (i.e., whether a local organization decides to deliver the new program), implementation (i.e., how well the program is conducted), and sustainability (i.e., whether the program is maintained over time) [44]. This paper outlines the ways in which we practically executed these processes. The implementation outcomes assessed during this project included: (1) adoption/stakeholder engagement, (2) fidelity, (3) provider satisfaction, (4) participant engagement, and (5) sustainability [2].

**Figure 3:**
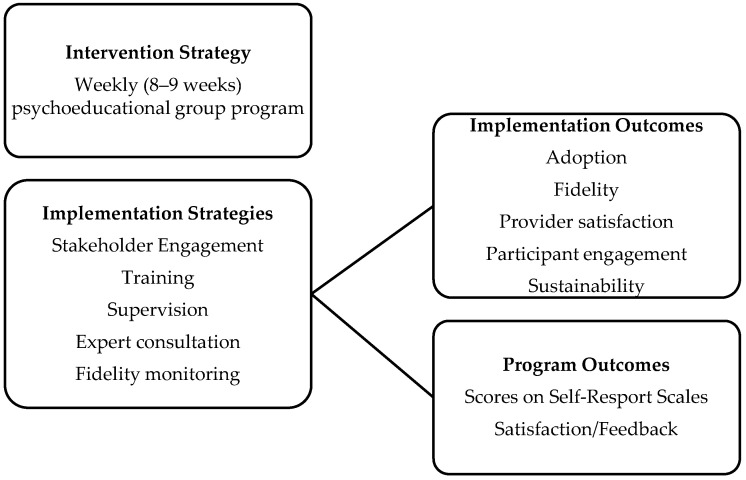
Implementation model.

This program is an example of a complex intervention in terms of the range of targeted skills, the expertise required by the facilitators, the number of program groups and hospital campuses, and the permitted level of flexibility of the intervention (i.e., fidelity) [26]. As such, to translate this mental health intervention into routine practice, an implementation model that involves a multi-strategy approach was adopted along with a fidelity monitoring framework [2,45]. Figure 3 illustrates the implementation model used within the project. By using this multi-strategy approach to implementation proposed by Powell et al., alongside structured fidelity monitoring guided by Carroll et al., the implementation of this program aligned with best practices recommended in the literature to support both its integration into practice outside of a research study and its effectiveness to achieve its expected results [2,45].

#### 2.4.1. Fidelity

Implementation fidelity is the degree to which programs are implemented as intended by the program developers [45]. Simply, staying true to the program that was studied helps achieve the intended outcomes and effectiveness of the intervention [45]. Carroll et al. provided a conceptual framework for implementation fidelity that consists of adherence in terms of content, coverage, frequency, and duration, as well as monitoring moderators, including intervention complexity, facilitation strategies, quality of delivery, and participant responsiveness [45]. Manualizing the program, along with significant training, is important for implementation fidelity. However, it is also necessary to apply methods that allow some flexibility and adaptation tailored to local contexts [46]. This project focused on fidelity related to adherence to the studied program (i.e., content and dose), quality of delivery, and participant and provider responsiveness [45].

#### 2.4.2. Implementation Strategies

**Figure 4:**
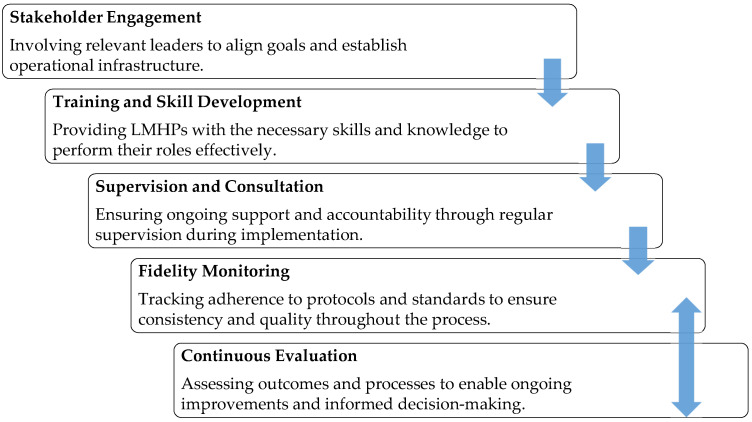
Implementation steps and strategies.

Implementation strategies are described as systematic steps to adopt and integrate evidence-based mental health innovations into routine care [47]. Powell et al. emphasized that using a variety of empirically supported implementation strategies, such as training, access to supervision, expert consultation, peer support, and ongoing fidelity monitoring is important for ensuring successful implementation and intervention sustainability [2]. RISE implementation applied a blended approach that incorporated all of these strategies packaged as a protocolized yet dynamic process. Figure 4 describes the implementation strategies utilized throughout the project.

Ensuring strict standardization of delivery is unrealistic as it involves a therapeutic dynamic and providers bring their own style, approach, and personalities to the delivery. Additionally, each group has its own “personality” and unique setting in which the program is delivered. Although ESIs adhere to strict protocols, it is important to allow room for a program provider’s clinical judgment, autonomy, strengths, and style of facilitation [46]. An intervention works better if a degree of adaptation is permitted [25].

#### 2.4.3. Ethical Considerations

RISE is a voluntary virtual psychoeducational group program. Informed consent was obtained from all participants, following guidelines outlined in legal and ethical mandates [48,49]. Participants were informed of confidentiality limitations before consenting to the program, and the facilitators provided guidelines for online group participation to safeguard privacy (e.g., ensuring they were in a private space before logging on to the session).

The program was delivered virtually using an online videoconferencing platform, Microsoft Teams, which is HIPAA compliant. The RISE group facilitators possess both technological and clinical competence to deliver online psychoeducation as outlined in the code of ethics for the American Counseling Association [48] and National Association of Social Workers [49]. The facilitators adhered to legal and ethical requirements for documentation and record keeping. Documentation for the program included a signed informed consent form, pre-group screening form, session process notes, completed fidelity rubric, and consultation notes if additional action was taken (e.g., referral).

The privacy of participants was a main priority, emphasized during every step of the implementation process. Confidentiality and its limitations were reviewed in detail during the informed consent pre-group screening. Only program facilitators and research personnel had access to participants’ personal information, which was clearly outlined in the informed consent. All project records are stored in a password-protected folders. Participants were informed that their participation had nothing to do with their employment, their privacy would be protected from leaders, and program participation required their commitment to the privacy of other group members.

### 2.5. Data Analysis

The quantitative data collected were analyzed for two separate groups, direct care nurses (n = 24) and nurse leaders (n = 26). Data analysis was performed using SPSS 28 software (IBM SPSS Statistics for Windows, version 28.0, IBM Corp., Armonk, NY, USA) at 0.05 alpha levels. Continuous variables were expressed as means and standard deviations, and categorical data were summarized as frequencies and percentages. Wilcoxon tests were used to test the difference between pre- and post- results. A Wilcoxon Rank-Sum test was conducted to test the differences between nurse leaders and direct care nurses on all measures except post-traumatic growth.

Analyzing feedback and perceived benefits of the intervention involved collecting and summarizing participant responses to an anonymous feedback form, and reporting the themes based broadly on techniques described by Braun and Clarke (2006) [50].

## 3. Results

### 3.1. Implementation Outcomes

#### 3.1.1. Adoption and Stakeholder Engagement

The successful implementation of the RISE program started with assessments of the setting and context of the target hospital campuses. The program aligned with these campuses’ executive strategic plans and committees, prioritizing workforce well-being and retention of high turnover employees, including nurses. During the 12-month pre-implementation phase, the research team, including the program developer, promoted the program through presentations to the executive nursing council, meetings with departmental leaders, and information sessions with clinical nurse leaders and educators. New partnerships were formed with campus CEOs and directors. The hospital leaders were provided with program materials and publications to allow them to learn about the core components, explore adaptation needs for their local context and culture, and assess operational infrastructure [47,51].

Each campus employed an LMHP who would be trained as the program facilitator. These LMHPs were already providing mental health support to employees through debriefs after adverse events, referrals, one-on-one support sessions, and educational workshops. Their established connections positioned them to advocate for the program and deliver it as an additional service for nurses and unit-based nurse leaders. The healthcare system’s research institute provided support services (i.e., regulatory oversight, data management, recruitment and enrollment guidance, cost estimation) to accommodate the operational demands of the project.

#### 3.1.2. Context and Operations

The organizational culture was supportive of staff self-care and psychological support, especially in the post-pandemic context. However, implementing the RISE program outside of a research study required building operational infrastructure to ensure effective marketing strategies, fiscal support, and personnel to facilitate reach and uptake. A multi-departmental operations team comprised of the program developer, research staff, mental health team manager, and nursing educators was formed to manage logistics. Collaborators included marketing and branding employees, clinical nursing leaders, a research scientific director, and campus C-suite leaders. Dedicated marketing efforts were needed to reach nurses and unit-based nurse leaders. Hospital leaders with influential and positional power—Chief Nursing Officers and nursing directors—instructed organizational marketing teams to promote the program via company newsletters, email listservs, flyers in break rooms, announcements at staff meetings, and outreach in new-hire orientation.

Only a nominal budget was required (e.g., participant workbooks), usually covered by the campus’s nursing budgets. The primary cost of running the program was facilitator time. Employed LMHPs delivered RISE sessions as part of their salaried roles. Leadership approval was essential to protect facilitator time for program duties. This approach was feasible, but at times put a burden on the facilitators who not only led the groups but also managed registrations, scheduling, and other administrative tasks. Advocacy is underway to fund a dedicated full-time program coordinator to manage administrative aspects in the future.

#### 3.1.3. Recruitment and Enrollment

The operations team established a rolling enrollment schedule, launching one nurse group and nurse leader group about every two months in 2023 and 2024, with a variety of days and times. This allowed potential participants to plan their work schedules accordingly to attend the weekly group sessions. Interested individuals registered via a Microsoft Forms link or QR code that was included in marketing materials. After signing up, each individual was contacted to schedule an introduction meeting with a facilitator. In this virtual meeting, the facilitator explained the program’s goals and commitment, obtained informed consent, discussed confidentiality and any risks, and assessed the nurse’s goals, level of motivation, and readiness. This screening step ensured the program would be a good fit for a participant before officially enrolling them into an upcoming group.

#### 3.1.4. Participant Engagement

Between the initial rollout in January 2023 and the last data collection timepoint in December 2024, 160 participants completed the RISE program. In the first year, attrition was a challenge, as many individuals who initially registered did not start or complete the program. Only about 19% of direct care nurses and 66% of nurse leaders of those who registered completed the full program. However, most attrition occurred between registration and group start due to work schedule conflicts or unknown personal reasons. While there was noticeable expressed interest in the program, efforts to address dropout after initial registration and lack of response after completing registration form were taken.

To improve engagement and retention, the team introduced attendance incentives, such as continuing education units (CEUs) and professional excellence points and began offering a variety of program days and times. Notably, time slots were made available to those who worked the night shift. Additionally, the facilitators developed a registration checklist to standardize follow-up procedures, including an introduction email and email and text reminders as the start date approached. They also started a waitlist as a way to fill potential withdrawals in upcoming groups. In 2024, retention rates improved for direct care nurses, with 41% of those that registered completing the program. Unit-based nurse leader retention remained relatively consistent, with 60% completing the program.

Word-of-mouth strategies were effective at reaching the “late majority” [52], and anonymous participant quotes were added into marketing materials to highlight the positive impact of the program. Among those who completed the feedback form, 93% of participants responded with a 10 out of 10 to “How likely are you to recommend RISE to a friend/colleague or leader?”.

#### 3.1.5. Fidelity

Implementation involved the following methods to ensure fidelity to the evidence-based program: (1) a three-day (24 h) live facilitator training led by the program developer, (2) use of a standardized program manual and facilitator guide, (3) use of program materials including a slide deck and handouts, (4) weekly supervision meetings with the developer, (5) use of a facilitator fidelity rubric, and (6) program improvement processes.

Quality of delivery was ensured through extensive training, supervision, and ongoing consultation from the developer [46,53], who is a LMHP, research scientist, and qualified clinical supervisor. Given the therapeutic component of the program, RISE facilitators are required to be LMHPs. Facilitators need a foundation of experience, knowledge, and skill to simultaneously manage group dynamics, teaching, emotional processing, and interpersonal processes.

##### Training

Eight trainees attended the RISE facilitator training. To ensure that the facilitators were prepared and met competency expectations, the three-day live training fused education through didactic content, interpersonal learning through discussion and reflection prompts, and skill development through experiential activities. It covered three broad objectives: (1) knowledge of group theory and approaches, (2) learning through reflection activities, role plays, and practice sessions, and (3) integration of personal therapeutic orientation with fidelity to the program [2,53].

Education was provided about the nursing profession (i.e., stressors, burnout), psychoeducational groups (i.e., ethics, developmental stages of groups, discrete techniques), the RISE program (i.e., theoretical framework, content, delivery methods), and program operations (i.e., recruitment, screening, fidelity, evaluation). Trainees were provided with a digital toolkit containing all of the materials, including a program manual and facilitator guide, informed consent template, RISE participant workbook, participant handouts, slide deck, pre-group screening form, group process note template, RISE fidelity rubric, and RISE feedback form.

To continuously improve training experience and outcomes, an anonymous voluntary feedback form was completed after the training. Trainees were asked if the following training components were helpful: learning, self-reflection, active discussion, group knowledge (i.e., theory, development, facilitation), skill development, and peer support. All respondents (n = 8) reported “yes” for each training component. Using a 5-point Likert scale, trainees evaluated the training and trainer. Results are shown in Table 1.

Feedback gathered through open-ended prompts included gratitude for learning from fellow mental health professionals, increases in their own insight and areas for growth, and improvement in clinical skills to apply when working with participants. Feedback for improvement included a suggestion to make some of the material on theory and background available as a web-based course.

##### Program Adherence

Program adherence, a primary measurement of fidelity, focused on the two main areas of content and dose [45]. Content is the skills or knowledge that the intervention seeks to deliver to participants. Program facilitators utilized materials (i.e., the slide deck), a standardized program manual and facilitator guide, and a facilitator fidelity rubric, which were all manualized in the studies. Dose consists of coverage, frequency, and duration [45]. All RISE group deliveries adhered to dose requirements established in the research, which involved weekly, 90-min synchronous group sessions with six to eight members per group. RISE for Nurse Leaders is nine weeks, and RISE for Direct Care Nurses is eight weeks. Group participants were encouraged to commit to attending all sessions during the informed consent meeting, though three absences were permitted before withdrawal from the program. This attendance threshold was established during the studies [28,31].

##### Fidelity Rubric

The facilitator fidelity rubric serves as a guide and check list to the delivery of the sessions. It is intended to be completed by the facilitator after each session and reviewed during each supervision meeting to evaluate adherence, performance, and areas for growth. The rubric outlines a checklist of content and activities that must be covered during each session, record of dose (i.e., length of session, participation, attendance), and self-assessment of delivery. Facilitators completed a self-assessment using a Likert scale to gauge their use of group techniques, therapeutic alliance, and the quality and use of process questions. This self-assessment served to maintain “quality of delivery,” which is outlined as a core component of measuring fidelity by Caroll and colleagues [45]. Rubrics were documented and then reviewed during each supervision session to evaluate and note any intervention drift that may have occurred.

##### Supervision Plan

The supervision plan was developed based on group counseling best practice [54,55], professional codes of ethics [48,49], and a counselor supervision development model [56]. After facilitators completed the training, they attended weekly supervision via videoconferencing with the developer during their first program delivery. If remediation was required, the developer constructed a plan to enhance skills and offered ongoing supervision. The inclusion of ongoing consultation significantly influences therapist adherence and skill [53], so this approach was built into the protocol of the implementation strategy.

#### 3.1.6. Provider Satisfaction

It was crucial to maintain a continuous feedback loop between program facilitators (i.e., providers) and the developer to ensure ongoing competency, well-being, and satisfaction. This process was accomplished through training feedback, bi-annual focus group meetings, and consultation and education. The bi-annual focus groups allowed facilitators to receive peer support, process any barriers, and explore needs. Overall, the facilitators reported satisfaction with the program itself and derived a sense of purpose and meaning from their role (e.g., “What I like most is having the opportunity to watch participants gain insight and new skills,” “This fills my cup”). The main barrier they noted was the added administrative burden of coordinating and managing the program (e.g., tracking registrations, scheduling). In response, efforts were made by the research staff to provide additional support related to marketing, streamlined registration, and participant workbook delivery.

#### 3.1.7. Sustainability and Adaptation

With continuous program enrollment, RISE continues to be a mental health support service to nurses and unit-based nurse leaders in the healthcare system. Several steps were taken to ensure the long-term sustainability of the program. An operations manual was created to standardize project administration. The nursing education department took on responsibility for managing CEUs and professional excellence points. There are summary reports on program utilization and impact provided to hospital leaders. Facilitators continue to receive ongoing training, education, and support, and they stay in continuous communication with the program developer through a Microsoft Teams group [2,57,58]. All program materials are updated with current evidence and saved in a private OneDrive folder.

Feedback gathered from facilitators and participants allowed ongoing adaptations to program delivery and implementation to improve its fit in this context [51,58]. While content and dose remained consistent, some changes to facilitation strategies were made based on participant requests, such as the use of virtual breakout rooms for deeper reflection and disclosure and more handouts and reading materials. Specific days and times were requested by participants, which were accommodated for future group offerings (e.g., early morning for the night shift).

### 3.2. Participant Outcomes

#### 3.2.1. Pre-Post Results

The demographic characteristics of the direct care nurses and unit-based nurse leaders who participated in the evaluation project are summarized in Table 2. The majority of participants work on medical-surgical, progressive care, and critical care units.

In the sample of direct care nurses, there were statistically significant improvements between pre-program and post-program scores in insight, self-compassion, self-efficacy (i.e., empowerment) and statistically significant reductions in burnout (i.e., emotional exhaustion) and perceived stress, as shown in Table 3. Although resilience scores improved between the beginning and end of the program, there was not a statistically significant difference in these scores (*p* = 0.051). Medium effect sizes (between 0.30 and 0.50) were seen in self-reflection and insight, perceived stress, and self-efficacy, with a small to medium effect size (less than 0.50) for self-compassion and emotional exhaustion.

For the nurse leaders’ sample, there were statistically significant improvements between pre-program and post-program scores in resilience, insight, self-compassion, and post-traumatic growth and statistically significant reductions in perceived stress, as shown in Table 4. Although the scores in burnout (i.e., emotional exhaustion) were lower after the program, there was not a statistically significant difference in these scores (*p* = 0.054). Small to medium effect sizes (less than 0.50) were seen for resilience, insight and self-reflection, self-compassion, and perceived stress, and a large effect size (greater than or equal to 0.50) was seen for post-traumatic growth.

A Wilcoxon Rank-Sum test was conducted to test the differences between nurse leaders and direct care nurses for BRS, SRIS-SF, Insight, Self-Reflection, SCS-SF, PSS, EE, DEP, and PA, and no significant differences were found for both pre- and post- measures.

#### 3.2.2. Participant Satisfaction

Among those who completed the program, 83 individuals (52%) completed feedback forms, most of whom were nurse leaders (75%). Overall satisfaction with the program was high (e.g., “I will never forget the impact this program has made in my life! Thank you from the bottom of my heart”). The following program components were rated as most helpful by respondents: learning and self-reflection (94% of respondents), mindfulness practice (93% of respondents), and active discussion and peer support (82% of respondents). The themes of the open-ended feedback prompts are summarized in Table 5.

Feedback for improvement generally related to program options, such as more variation in group times and dates and in-person groups. For example, a respondent stated, “I work nights, and the schedule was really hard to keep. After 3 p.m. is good timing.” Several respondents reported that marketing and advertising efforts should be expanded so that more people could have access to the program.

#### 3.2.3. Quality Management

A quality improvement project is ongoing to continuously assess program outcomes for individual participants, integrate feedback for improvement, and maintain enrollment and retention goals. Goals to maintain the program’s quality include (1) analyzing pre-post data from validated instruments annually, (2) enhancing recruitment activities, (3) gathering participant feedback after each group, (4) reporting results to stakeholders and executive leaders, (5) supporting facilitators through bi-annual focus groups and continuing education, (6) monitoring fidelity, and (7) making changes to the program and its implementation based on evaluation outcomes.

## 4. Discussion

RISE is an evidence-based psychoeducational group program for nurses and unit-based nurse leaders that can be integrated into the organizational approach of healthcare systems as one part of a comprehensive strategy to support mental well-being [27,59]. This paper describes the process for implementation in a multi-campus healthcare system to show how it can be a scalable program with positive effects on well-being.

### 4.1. Enabling Factors of Implementation

An underlying factor facilitating the successful implementation of the RISE program is broad concern about the prevalence and harmful consequences of burnout and psychological suffering in nurses. Leaders acknowledged that this program is relevant, promising, and consequential to their teams, which is an important dynamic in diffusion and uptake [52]. Support from nursing leadership and other stakeholders was crucial in scaling RISE as an embedded resource. Buy-in and engagement occurred at multiple levels, including executive leadership for financial support, clinical leadership to cultivate a permission structure, program operations to ensure infrastructure was in place to support recruitment efforts and management, qualified partners to deliver the intervention, and program users (i.e., nurses) [2,25].

Another enabling factor is the increased demand for empirically based methods to treat and mitigate burnout and other stress-related injuries. RISE filled a need for programs that go beyond prevention and education to promote healing and recovery. For example, mobile-based or other digital educational services may be useful in prevention efforts and are easier to scale-up [19]. However, these approaches may not be effective as a treatment approach for those already experiencing burnout. Evidence shows that cognitive–behavioral and mindfulness interventions have moderate effects on stress injuries, though many related studies have design limitations and vary widely in duration and protocols [19,60]. Furthermore, the literature supports the therapeutic underpinning of RISE (i.e., mindfulness, CBT, ACT) as effective in addressing stress, well-being, and burnout [21,60,61,62,63,64].

An important and positive feature of the RISE program is that it is delivered by LMHPs to ensure best practice and application of therapeutic strategies. Recent literature supports the use of qualified professionals over volunteers or peers [65,66]. LMHPs can ensure expertise, continuity of quality care over time, and adherence to legal and ethical guidelines [66]. Training these LMHPs to be effective, qualified providers was key to successful implementation. The literature consistently identifies multi-faceted training approaches as the most effective, which include multiple days of immersive training, expert consultation, a treatment manual, review of sessions, supervision, and behavioral rehearsal [53,58,67]. The RISE training adhered to these recommendations and involved a process that was dynamic, active, and addressed a variety of learning styles.

Synchronous virtual delivery through a videoconferencing platform was used previously in the research studies and currently during this operationalization of the program. This feasible approach makes the program accessible and convenient for busy healthcare professionals. Current literature supports virtual delivery as an effective strategy [60]. Participants reported feeling connected to and supported by each other in this format, which is consistent with other studies involving online group interventions [68,69].

Fidelity to the core components of the program was also crucial for implementation and positive outcomes. The key is to be clear about how much adaptation is permissible and to note intervention drift so that fidelity can be re-established based on the protocol [25]. Appropriate implementation criteria were established during the studies and through fidelity methods such as rigorous training and supervision by the developer, a manualized curriculum and operations guide, and an ongoing feedback loop between the developer, providers, and local stakeholders [2]. Facilitators stayed true to the program components, content, and theoretical framework, while incorporating their own therapeutic style and personality during delivery. Adherence to content and dose was consistent and likely contributed to positive participant outcomes [2,45].

### 4.2. Participant Outcomes

Based on participant satisfaction reports and quantitative results, RISE shows promise as an impactful resource in real-world settings. Statistical analyses showed improvement with small to large effects in well-being indicators associated with the RISE themes of resilience, insight, self-compassion, and empowerment/self-efficacy.

There were also reductions in perceived stress and burnout (i.e., emotional exhaustion), though changes in the burnout scores for leaders were not statistically significant (*p* = 0.054). This is likely related to lower scores at baseline or small sample size rather than a reflection of an inappropriate aim or measure. RISE as an operationalized service does not have inclusion criteria, which enables it to reach all nurses who desire help and support, rather than only those who have a specific set of symptoms (e.g., high burnout). Moreover, offering individual-level interventions without structural changes in the work environment is not sufficient in addressing the burnout crisis. Offering RISE in conjunction with environmental changes such as adequate staffing, supervisory support, and efficient workflow may lead to greater and more sustained improvements in burnout among nurse leaders [30,31].

Similarly, resilience in direct care nurses improved, though the changes in scores were not statistically significant (*p* = 0.051). We suspect that with a larger sample size, it would likely reach significance, as was evidenced in the randomized controlled trial. Nurses, in general, may possess adaptive coping skills due to challenging training and preparation, leaving less room for improvement in resilience scores from baseline. This could potentially explain the lower efficacy of interventions targeting resilience due to a potential ceiling effect, for example [70].

For unit-based nurse leaders, post-traumatic growth, the primary outcome within the studies, significantly improved with a large effect size, indicating a need for continued exploration into traumatic stress among this population and the usefulness of RISE to address this concern. The ramifications of the pandemic revealed a necessity for services that extend beyond burnout prevention to include broad mental health support to address trauma in the workforce [16]. For example, work in hospitals exposes employees to extremely distressing events, such as patient suffering, harmful treatments, death, and workplace violence [16,71]. This program can facilitate post-traumatic growth through mindful self-care, sharing painful emotions, and processing internal experiences with others who have shared adversity [72,73,74].

The most consistent findings from participant feedback were related to improvement in insight, self-awareness, and self-compassion. Participants consistently reported that they acquired skills to explore their thoughts, behaviors, and emotions, and expressed a desire to be kinder to themselves (see Table 5). Quantitative results support this with significant changes in scores in self-compassion and insight for both direct care nurses and nurse leaders. Participants noted that program attendance empowered them to make changes in their daily life related to three main themes: mindfulness practice, self-compassion, and dedicated time for self. Themes related to the impact of the program on well-being were improved coping skills, new perspectives and insight, and social belonging. The literature supports the statement that healthy coping behaviors, resilience, and social support are associated with positive psychological outcomes [75], and social support in particular protects the mental health of nurses [76,77]. Participants reported that the program provided needed connections, which offered relief from isolation, learning, and validation of experiences.

### 4.3. Factors Leading to Implementation Challenges

Through quality improvement processes, program management was identified as an implementation barrier for the operations team. As one facilitator reported, “so much effort must be put in at the beginning to market, spread the word, and get team members to sign up. The calls and emails back and forth and taking time to respond” were difficult. Implementation studies of mental well-being interventions show that there is often no managerial support to prepare or deliver programs within an organization [78]. In fact, providers report delivering these programs in addition to their expected job duties [78]. This is consistent with our findings that scaling the RISE program is feasible in the hospital setting with the necessary personnel to dedicate time and effort. Additionally, hospitals may not employ LMHPs and therefore may contract external licensed professionals to deliver the program. Advocacy continues for a dedicated full-time employee to manage program operations.

Though outcomes are generally positive, interventions like the RISE program are time-intensive and require a personal investment [20,21,79]. Because participation in the program is voluntary, success depends on engaging interested participants in the early and late majority phases of diffusion [52]. An incentive structure, including offering continuing education units (CEUs) and professional excellence points, can enhance engagement and retention in the program. Participant engagement strategies utilized in this project included virtual delivery for ease of access, offering several scheduling options, an emphasis on confidentiality, word-of-mouth strategies, and providing private screening sessions with an LMHP for a full informed consent process.

### 4.4. Scaling and Fidelity Monitoring

Scaling evidence-based treatments for mental well-being requires qualified providers to deliver programs with high fidelity [80]. Given the need for a scalable solution in healthcare systems, a train-the-trainer (TTT) model was adopted, which can enhance provider capacity and facilitate widespread diffusion [58,80]. This model involves the development of a trainer who then trains and supervises LMHPs in their respective settings and serves as an internal coach. The trainer is initially coached by experts (i.e., the program developer), who continue to provide long-term consultation and supervision throughout the implementation process [58,80]. Trainers are then equipped to train RISE facilitators, who deliver the program for their nursing population. With a focus on quality of delivery and adherence to the program, this project illustrates a strategy to sustain fidelity through preparing effective LMHPs. Also embedded within the training curriculum are skills and strategies to build buy-in and cultivate visible support from hospital leadership, which are crucial steps in dissemination and diffusion [52].

### 4.5. Economic Considerations

Healthcare systems must invest in the well-being of their nurses to enhance outcomes and ultimately improve quality of care and patient safety. Prior studies have shown that, for every dollar invested in employee wellness, the return on investment is $3 to $4.48 [21]. The value of investment also increases because staff perceptions of organizational support can itself positively impact mental well-being [21,81].

Economic considerations were gauged during the stages of intervention development, study, and implementation. Financial investment in the development and study of the RISE program was supported by the research department through funding sources. Having established protocols and evidence of feasibility during the studies, most of the cost to operationalize this program was related to facilitator time, with a nominal budget for program materials. Leaders protected provider time, rather than compensating them in addition to their salary. If a healthcare system does not employ qualified LMHPs, an additional expense would include contracting these individuals.

Economic analyses reported to decision makers can affect their buy-in and whether and how they invest in programming [26]. Sawyer and colleagues conducted a retrospective cost analysis after the first RISE study [29]. Cost savings were calculated among nurses who completed the intervention compared with matched control subjects. Turnover cost per RN and program costs per participant who attended the intervention (n = 64), were calculated. Given the reduction in turnover by 20%, this calculation showed the net cost savings by the healthcare organization to be $574,800 for this one study [29].

Economic considerations also relate to an incentive structure to enhance reach and uptake. Participants in this project attended group sessions on their own time and perceived this program as a personal growth and recovery experience they felt was worth the time investment. Enabling nurses to clock in during sessions might further improve utilization and retention and reinforce a culture that supports the mental health of its workforce. Current literature shows that common barriers to participation in workplace interventions among healthcare workers were inadequate staff, high workload, time pressures, work constraints, lack of manager support, scheduling health programs outside work hours, and lack of motivation [79]. It is suggested that workplace interventions be implemented as routine offerings with free work hours to encourage participation or integrate intervention activities into daily operations [79]. Given the estimated costs associated with burnout [13], it seems evidence-based treatments for burnout and mental health are a worthy investment for healthcare systems.

### 4.6. Limitations

While attempts to measure participant outcomes over time were made, there was some attrition at the one-month data collection timepoint, which prevented the determination of the sustainability of outcomes. Incentives, which were used in the research studies, may be offered to participants in the future to encourage survey completion at the three-month and six-month follow-up timepoints. As with any voluntary psychosocial intervention, self-report is a standard method of outcome measurement. This introduces the possibility of social desirability bias, which involves the tendency of respondents to answer survey questions in a favorable way. Also, individuals who choose to sign up for the program may have a predisposition for personal growth and help-seeking, which could potentially indicate a self-selection bias. Lastly, the lack of a control group and the small sample size in this QI/QA project limits interpretation of the results related to causality and generalizability.

### 4.7. Future Research

Future research could assess the cost effectiveness of specific implementation strategies for this program. Also, systemic outcomes, such as unit-level metrics and retention rates, could be measured and analyzed for those who have attended RISE compared to those who have not. Additionally, new studies are underway by other research teams to further test generalizability of the RISE program as an empirically supported intervention (ESI). A specific population of interest includes RISE for informal caregivers of persons with dementia or other cognitive impairments.

### 4.8. Implications for Healthcare Leaders

Offering programs such as RISE can be one part of a broad strategy to help support nurses and protect their mental health. A variety of different approaches are needed for workplace mental health interventions that range from prevention to psychological treatment to changes in the work environment [19,82]. Secondary and tertiary interventions (i.e., individual-level programs), like RISE, can treat or mitigate symptoms of distress [19], while primary interventions aim to change organizational structures that cause stress injury (e.g., flexible scheduling) [82]. Organizational efforts should aim to combine structural changes in the practice environment with individual-level interventions. For example, lower workloads and healthier work environments are associated with reduced risks of burnout [83,84].

It is imperative that healthcare organizations prioritize the mental health and well-being of nurses and provide effective programming that cares for them. As a multidimensional intervention that combines education with therapeutic process, RISE addresses the distress experienced by nurses in today’s complex healthcare environment, and it can be scaled within a hospital system. RISE can be implemented and tested in non-acute care settings as well, following the strategies outlined in this paper.

## 5. Conclusions

This project evaluated the implementation and impact of an evidence-based intervention outside of a research study. This process balanced fidelity to the original program with necessary adaptations to local contexts, which are both critical for successful implementation. The RISE program demonstrates strong potential as a practical and effective solution within healthcare settings. Its implementation was made possible through key processes including stakeholder engagement at multiple levels of leadership, interdepartmental collaboration, accessibility, fidelity to the core components of the studied program, and rigorous training of mental health providers who ensure best practice and adherence to programmatic standards. Ultimately, this paper offers guidance to administrators and nursing leaders who are seeking to embed evidence-based mental health programming into routine employee care.

## Figures and Tables

**Table 1:** Training Performance Evaluation.

Trainer Evaluation Prompt	Trainee Response, n = 8
I was satisfied with the RISE training experience.	100% Strongly Agree
I feel equipped/have the tools to deliver the RISE program.	100% Strongly Agree
The trainer was knowledgeable of the content.	100% Strongly Agree
The trainer engaged me in the learning process.	100% Strongly Agree
The trainer answered my questions and addressed concerns.	100% Strongly Agree

**Table 2:** Participant Demographics.

Demographic Variable	Nurse Leaders (n = 26)		Direct Care Nurses (n = 24)	
	Count	Percent (%)	Count	Percent (%)
Age				
25–34	3	11.5	3	12.5
35–44	12	46.2	10	41.7
45–54	8	30.8	7	29.2
55–64	3	11.5	3	12.5
65 or older	0	0	1	4.2
Gender				
Male	1	3.8	2	8.3
Female	25	96.2	22	91.7
Race				
White	18	69.2	14	58.3
Black or African American	2	7.7	1	4.2
Asian	5	19.2	6	25.0
Multi-Racial/Multi-Heritage	1	3.8	3	12.5
Ethnicity				
Non-Hispanic or Latino/Latina	21	80.8	19	79.2
Hispanic or Latino/Latina	5	19.2	5	20.8
Years of Experience				
0–2 years	4	15.4	2	8.3
3–5 years	6	23.1	1	4.2
6–10 years	7	26.9	6	25.0
Over 10 years	9	34.6	15	62.5
Unit				
Clinical operations	1	3.8	0	0.0
Critical care	4	15.4	5	20.8
Emergency department	1	3.8	2	8.3
Medical-surgical	4	15.4	5	20.8
Mother-baby	0	0.0	1	4.2
Outpatient cardiac rehab	0	0.0	1	4.2
Progressive care unit	6	23.1	5	20.8
Peri-operative	2	7.7	2	8.3
Procedural/ambulatory	3	11.5	2	8.3
Rapid response	0	0.0	1	4.2
Inpatient rehab	1	3.8	0	0.0
Labor and delivery	2	7.7	0	0.0
Operating room	1	3.8	0	0.0
Surgery	1	3.8	0	0.0

**Table 3:** Results of Pre-Post-Analysis (Wilcoxon test) for Direct Care Nurses.

Variables	Pre		Post		Effect Size	Z Score	*p*-Value
	Mean	SD	Mean	SD			
BRS	20.54	4.77	21.88	4.19	−0.298	−1.955 ^b^	0.051
SRIS-SF	58.67	10.68	64.83	10.61	−0.579	−3.176 ^b^	0.001
Insight	28.46	6.90	32.13	6.95	−0.530	−3.029 ^b^	0.002
Self-Reflection	30.21	7.43	32.71	6.93	−0.348	−1.776 ^b^	0.076
SCS-SF	36.13	9.30	40.04	9.92	−0.407	−2.345 ^b^	0.019
PSS	17.25	4.11	14.63	5.07	0.568	−2.565 ^c^	0.010
EE	23.17	12.29	20.38	13.05	0.220	−2.323 ^c^	0.020
DEP	5.88	5.01	5.38	4.93	0.101	−0.787 ^c^	0.431
PA	37.88	6.74	38.17	7.31	−0.042	−0.070 ^b^	0.944
GSE	29.88	4.0	31.79	3.55	−0.505	−4.293 ^c^	0.000

^b^ Based on negative ranks. ^c^ Based on positive ranks. Note. BRS = Brief Resilience Scale; SRIS-SF = Self-Reflection and Insight Scale-Short Form; SCS-SF = Self-Compassion Scale−Short Form; PSS = Perceived Stress Scale; MBI = Maslach Burnout Inventory; EE = emotional exhaustion; DEP = depersonalization; PA = personal accomplishment; GSE = General Self-Efficacy.

**Table 4:** Results of Pre-Post-Analysis (Wilcoxon test) for Nurse Leaders.

Variables	Pre		Post		Effect Size	Z Score	*p*
	Mean	SD	Mean	SD			
BRS	21.15	3.55	22.50	3.82	−0.366	−1.973 ^b^	0.049
SRIS-SF	61.00	12.97	66.50	11.70	−0.445	−1.966 ^b^	0.049
Insight	28.77	8.34	32.50	7.49	−0.471	−2.941 ^b^	0.003
Self-Reflection	32.23	7.76	34.00	5.74	−0.259	−0.868 ^b^	0.385
SCS-SF	38.15	9.44	41.35	9.95	−0.330	−2.188 ^b^	0.029
PSS	16.69	6.05	13.88	6.72	0.439	−2.659 ^c^	0.008
EE	23.92	12.33	21.38	13.83	0.194	−1.927 ^c^	0.054
DEP	5.96	5.10	5.73	5.87	0.042	−0.803 ^c^	0.422
PA	39.69	4.94	40.50	5.19	−0.160	−0.593 ^b^	0.553
Meaning	6.18	0.74	6.18	0.84	0	−0.762 ^b^	0.446
Competence	5.87	0.74	6.03	0.87	−0.198	−1.214 ^b^	0.225
Self-Determination	5.45	1.13	5.68	1.09	−0.207	−1.241 ^b^	0.215
Impact	5.17	1.40	5.46	1.05	−0.234	−0.911 ^b^	0.362
PEI	5.67	0.87	5.84	0.85	−0.198	−1.459 ^b^	0.145
PTGI	49.80	32.81	68.64	29.31	−0.606	−2.772 ^b^	0.006

^b^ Based on negative ranks. ^c^ Based on positive ranks. Note. BRS = Brief Resilience Scale; SRIS = Self-Reflection and Insight Scale; SCS-SF = Self-Compassion Scale−Short Form; PSS = Perceived Stress Scale; EE = emotional exhaustion; DEP = depersonalization; PA = personal accomplishment; PEI = Psychological Empowerment Instrument; PTGI = Post-traumatic Growth Inventory.

**Table 5:** Themes of Open-Ended Participant Feedback Prompts.

Feedback Prompt	Response Theme	Representative Quotes
What was the impact of this program on your well-being?	Improved coping	I received valuable resources and tools for coping with life struggles personally and professionally. I’m more prepared to deal with difficult situations. My toolbox got bigger. I have learned and grown so much from this program in my personal and professional life.
	New perspective/insight	It was unlimited. It really brought so much insight on myself and how to be a better person. It made me think about myself and work and how I can handle adversity and challenges. It helped me understand myself better and to acknowledge (and put a name to) my feelings. I feel so much better about being myself and loving myself. Amazing…changed my perspective.
	Social belonging	It made me realize how I am not the only one experiencing what I am experiencing, and it gave me a sense of “it’s okay to not be okay.” I feel more supported as a leader knowing others have experienced the same things as I have. It was nice to join a group of nurses who have been in the field a long time. Sharing these experiences makes me feel that I am not alone.
Name at least one thing you plan to change in your daily life.	Mindfulness practice	Being mindful and present, taking time for me to be better for the team. Stop, notice, and observe. Validate feelings and then let them go. Incorporate the exercise of breathing. It makes a great difference. Mindfulness and calming myself Mindful breathing to recenter myself
	Self-compassion	Practice self-compassion and reflect each day on the good things from the day Patience, grace, and self-compassion Be more compassionate to myself Be kinder to myself, the importance of filling my cup so I can be there for others Learn to love myself better
	Dedicated time for self	Have some time for myself Take time for me even if it is five minutes Take time to reflect on the day Make self-care a priority

## Data Availability

The data presented in this study are available on request from the corresponding author due to privacy and ethical considerations surrounding confidentiality.
